# Calvarial osteoblast gene expression in patients with craniosynostosis leads to novel polygenic mouse model

**DOI:** 10.1371/journal.pone.0221402

**Published:** 2019-08-23

**Authors:** Jonas A. Gustafson, Sarah S. Park, Michael L. Cunningham

**Affiliations:** 1 Seattle Children’s Research Institute, Center for Developmental Biology and Regenerative Medicine, Seattle, Washington, United States of America; 2 Seattle Children’s Hospital Craniofacial Center, Seattle, Washington, United States of America; 3 University of Washington, Department of Pediatrics, Seattle, Washington, United States of America; Mayo Clinic Minnesota, UNITED STATES

## Abstract

Craniosynostosis is the premature fusion of the sutures of the calvaria and is principally designated as being either syndromic (demonstrating characteristic extracranial malformations) or non-syndromic. While many forms of syndromic craniosynostosis are known to be caused by specific mutations, the genetic etiology of non-syndromic, single-suture craniosynostosis (SSC) is poorly understood. Based on the low recurrence rate (4–7%) and the fact that recurrent mutations have not been identified for most cases of SSC, we propose that some cases of isolated, single suture craniosynostosis may be polygenic. Previous work in our lab identified a disproportionately high number of rare and novel gain-of-function *IGF1R* variants in patients with SSC as compared to controls. Building upon this result, we used expression array data from calvarial osteoblasts isolated from infants with and without SSC to ascertain correlations between high *IGF1* expression and expression of other osteogenic genes of interest. We identified a positive correlation between increased expression of *IGF1* and *RUNX2*, a gene known to cause SSC with increased gene dosage. Subsequent phosphorylation assays revealed that osteoblast cell lines from cases with high *IGF1* expression demonstrated inhibition of GSK3β, a serine/threonine kinase known to inhibit RUNX2, thus activating osteogenesis through the IRS1-mediated Akt pathway. With these findings, we have utilized established mouse strains to examine a novel model of polygenic inheritance (a phenotype influenced by more than one gene) of SSC. Compound heterozygous mice with selective disinhibition of RUNX2 and either overexpression of *IGF1* or loss of function of GSK3β demonstrated an increase in the frequency and severity of synostosis as compared to mice with the RUNX2 disinhibition alone. These polygenic mouse models reinforce, in-vivo, that the combination of activation of the IGF1 pathway and disinhibition of the RUNX2 pathway leads to an increased risk of developing craniosynostosis and serves as a model of human SSC.

## Introduction

Single-suture, non-syndromic craniosynostosis occurs in approximately 1/2500 live births with a familial recurrence rate of 4–7% [1, 2]. Typically, when an infant is born, the calvaria are separated by soft membranous tissue (intrasutural mesenchyme) to allow for skull deformation during birth and brain growth throughout childhood. Premature fusion of the sutures of the calvaria is associated with distortion of skull growth that can be associated with increased intracranial pressure due to constraint of the growing brain and asymmetric facial growth that can lead to dental and vision complications. Currently, the only treatment available for this condition is major, invasive surgery to expand the cranial vault.

A limited number of single gene mutations are known to cause single-suture craniosynostosis (SSC) (*FGFR3* [3, 4], *TWIST1* [4, 5], *EFNB1* [6] and *TCF12* [7]), but they are most often associated with syndromic features. Mutations in more than 60 genes have been described in syndromic forms of craniosynostosis [8]. These genes and their related pathways give us insight into the molecular functions that may be involved in its etiology. Given this information, and the known recurrence rate of SSC, we propose that many instances of SSC are caused by dysregulation of a combination of genetic pathways.

Several genes have emerged as potential polygenic candidates through our RNA microarray and expression analysis, genomic sequencing, phosphorylation studies, and biomechanical assays. We first performed targeted candidate gene sequencing on 186 patients with SSC and identified rare sequence variants in *IGF1R* that were overrepresented in an SSC case population [9]. Structural and functional analysis of these IGF1R variants suggested that they cause activation of the IGF1 pathway [9, 10], which is well known to be involved in osteogenesis. IGF1/IGFR1 signaling promotes osteogenic differentiation of calvarial osteoblasts and mesenchymal stem cells inducing expression of *COL1A2*, *ALP*, and *RUNX2*, and matrix mineralization [11–14]. Taken together, these data suggest that activation of the IGF1 pathway may predispose to the development of craniosynostosis.

Concurrently, our lab used correlation analysis of osteoblast transcriptomes from 199 human cases (including the 186 from the targeted sequencing project) and 50 controls to detect distinct patterns of gene expression that were associated with craniosynostosis [15, 16]. Two subpopulations emerged that were unique to cases and distinct from one another [10]. Comparison of these subtypes to existing expression profiles available through the Gene Expression Omnibus (GEO) identified that the expression patterns from our experiments were correlated with datasets specifically related to osteoblast mineralization [17]. Phosphorylation analysis of cell lines from these two subgroups, as well as cell lines with presumed gain-of-function *IGF1R* variants [10], demonstrated distinct activating phosphorylation patterns of the IRS1-mediated Akt pathway [15]. This activation of the Akt pathway lead to phosphorylation of GSK3β (Ser9), targeting it for degradation and resulting in disinhibition of RUNX2 and β-catenin [18], known positive regulators of osteoblast differentiation. The PI3K/Akt signaling pathway is a critical part of a complex network regulating bone development [19]. Although not well studied in membranous bone development, the Akt pathway is important in mesenchymal stem cell osteogenesis [20], osteoblast proliferation and differentiation [21], and chondrogenesis [22].

Subsequent biomechanical assays measuring contractility and migration were performed on primary SSC osteoblasts with and without elevated *IGF1* expression. These experiments revealed increased contractility and decreased migration in cells with elevated levels of IGF1, supporting our previous results suggesting that enhanced differentiation is correlated with this subset of SSC cases [23]. The identification of Akt pathway activation, cellular phenotypes consistent with enhanced differentiation in SSC osteoblasts demonstrating increased *IGF1* expression, and gain-of-function mutations in *IGF1R* made the IGF1/IGF1R pathway an obvious choice for further examination.

Using the same patient cohort, we performed copy number variant analysis and identified a duplication of *RUNX2* in two cases with familial metopic SSC and hypodontia [24]. This observation has now been validated by other groups reporting increased dosage of RUNX2 in families with craniosynostosis [25]. In addition to being a known positive regulator of osteoblast differentiation [26, 27], RUNX2 is known to be inhibited by TWIST1 [28, 29]. Mutations in *TWIST1* cause Saethre-Chotzen syndrome, a hereditary form of craniosynostosis [30, 31], however, we have shown that disinhibition of RUNX2 caused by mutations in the highly conserved TWIST1 twist-box domain is associated with non-syndromic unilateral coronal and sagittal craniosynostosis [5].

In this manuscript we present data from mouse cross-breeding experiments designed to explore possible polygenic inheritance of craniosynostosis using *Twist1*/*Igf1* and *Twist1*/*Gsk3β* compound heterozygotes to test hypotheses generated from our previous human expression data. These mouse models demonstrate an increased frequency and severity of craniosynostosis and support the hypothesis that combined dysregulation of the TWIST1 and IGF1 pathways could contribute to an increase risk in humans.

## Methods

### Ethics statement

This study was reviewed and approved by the Institutional Review Board of Seattle Children’s Hospital (approval number 12394). Written informed consent from parents or guardians of children with SSC was obtained and a consent waiver was obtained for the use of anonymous control samples. See reference [9].

### Calvarial tissue sample collection

Post-surgical calvarial bone samples which would have otherwise been discarded were collected from 211 cases and 50 controls between the years of 2002 and 2006. Computed Tomography confirmed the diagnosis of isolated sagittal, metopic, or unilateral coronal synostosis in each case. All cases were screened for the presence of major medical conditions such as syndromic craniosynostosis, cardiac defects, seizure disorders, cerebral palsy, health conditions requiring surgical intervention, presence of three or more minor extra-cranial malformations; or presence of any other major malformations. No individuals with these conditions were enrolled. Control samples were obtained from patients undergoing a craniotomy for reasons other than craniosynostosis (e.g. brain tumor, hydrocephalus, etc.) or autopsies. Control samples were not obtained from individuals with disorders that affect bone such as skeletal dysplasias. Tissue samples were collected in Waymouth’s Media (WM) (10% FBS, 2% Corning Antibiotic Antimycotic Solution 100X, Corning, NY) and sent to the lab for processing.

### Osteoblast growth

Calvarial tissue was rinsed in WM and the surrounding soft tissue was removed. Using a sterile scalpel blade, the tissue was cut into 1-2mm pieces and 2 pieces per well were cultured in 12-well plates at 37°C, 5% CO_2_, and 99% humidity. Upon confluence, cells were washed with PBS, trypsinized with 0.05% Trypsin-EDTA (GE Hyclone, Logan, UT), and passaged into T75 flasks. Confluent cells were cryogenically stored in freezing media containing 90% FBS and 10% dimethyl sulfoxide in a liquid nitrogen freezer. As previously descried, alkaline phosphatase activity was used to assure osteoblast lineage and all samples demonstrated high ALP activity [32].

### RNA extraction

Cell lines were cultured to sub-confluence in T25 flasks and passaged to a density of 175,000 cells per 25cm^2^. At 75% confluence, cells were trypsinized, washed in cold 1X PBS, and RNA was isolated using the Roche High Pure miRNA Isolation Kit (Roche, Indianapolis, IN) in accordance with the manufacturer’s instructions. Initial thawing of the cells for this process was done in batches, and batch effect was considered during expression analysis. RNA integrity was assessed using the Agilent 2100 Bioanalyzer and only samples with a RIN score of >8.0 were used for further analysis.

### cDNA microarray and expression analysis

cDNA microarray assays were performed on 261 calvarial tissue samples (211 cases and 50 controls) using Affymetrix Human Gene 1.0 ST array technology. See full methods in reference [15].

All microarray data are MIAME compliant and the raw dataset has been deposited in the MIAME compliant Gene Expression Omnibus (GEO) database under accession number GSE27976 [17]. For the purposes of this study, transcript expression analysis was restricted to 399 transcripts (S1 Table) chosen *a priori* based on their role in bone development and osteoblast differentiation. Transcript expression levels were assessed visually by applying conditional formatting to expression level values for each gene and analyzed by paired t-test.

### Mouse husbandry, genotyping, and phenotyping

All mouse work was approved by Seattle Children’s Institutional Animal Care and Use Committee and complies with NIH Guidelines for the Care and Use of Laboratory Animals. Commercially available mouse lines *Igf1*^(+/tg)^/*Igf1*^(tg/tg)^ (transgenic overexpression of *Igf1*), *Gsk3β*^(+/-)^ (hemizygous knockout of *Gsk3β*), and *Twist1*^(+/-)^ (loss-of-function point mutation in *Twist1* “twist-box” domain) from Jackson Laboratories, Bar Harbor, ME were bred to congenicity on a C57BL/6J background with a minimum of 9 backcross generations (≥99.5% identity). See official Jackson Laboratory nomenclature (S2 Table). Compound heterozygous mice were generated from crossing these three strains to one another (Table 1). The genotype of each mouse was determined by PCR for *Gsk3β*^(+/-)^ and *Igf1*^(+/tg)^, and by Sanger sequencing for *Twist1*^(+/-)^. Primers are listed in S3 Table. Phenotyping for polydactyly was used to determine Twist1 heterozygosity once congenicity was established as it is a fully penetrant trait [33]. Homozygous *Twist1* and *Gsk3β* mice are known to be embryonic lethal and thus were maintained as heterozygotes [34, 35]. *Gsk3β*^(+/-)^ mice have been shown to have significantly lower *Gsk3β* expression than their wildtype counterparts [36] *Igf1* mice were maintained as both homozygotes and heterozygotes. Mouse IGF1 ELISA Kit (Abcam, ab100695, Cambridge, MA) was used to validate overexpression of IGF1 in the serum of IGF1 transgenic animals (reported by Wu, et al [37]). Random sampling of 4 transgenic and control mice revealed a greater than 3-fold increase in expression of IGF1 (20.6±3.8 vs. 6.0±2.3 pg/ml, respectively). Because neither the twist-box point mutation nor the disinhibition of RUNX2 can be quatified by protein or RNA expression, we inferred the effect of the *Twist1* missense mutation on RUNX2 by observation of the polydactyly phenotype in *Twist1*^(+/-)^. mice. Litters of interest were sacrificed at P28 by CO_2_ euthanasia and prepared for microCT scanning.

**Table 1:** Genotypes of mouse crosses performed and subsequent pups of interest.

Parentage	Pups of interest
C57BL/6J x C57BL/6J	C57BL/6J
*Igf1*^(+/tg)^ x C57BL/6J	*Igf1*^(+/tg)^
*Igf1*^(+/tg)^ x *Igf1*^(+/tg)^	*Igf1*^(tg/tg)^
*Gsk3β*^(+/-)^ x C57BL/6J	*Gsk3β*^(+/-)^
Twist1^(+/-)^ x C57BL/6J	*Twist1*^(+/-)^
*Gsk3β*^(+/-)^ x *Igf1*^(+/tg)^ (or *Igf1*^(tg/tg)^)	*Gsk3β*^(+/-)^/*Igf1*^(+/tg)^
*Twist1*^(+/-)^ x *GSK3β*^(+/-)^	*Twist1*^(+/-)^/*Gsk3β*^(+/-)^
*Twist1*^(+/-)^ x *Igf1*^(+/tg)^ (or *Igf1*^(tg/tg)^)	*Twist1*^(+/-)^/*Igf1*^(+/tg)^

### MicroCT scanning and image rendering

A Skyscan 1076 micro Computed Tomography (microCT) scanner was used to render high-quality 3-dimensional CT images of skulls from P28 mice with our genotypes of interest. Scans were performed at a maximum of 35um slice thickness and with the following parameters; 55Kv, 179uA, 0.5mm aluminum filter, 360 ms exposure, rotation step of 0.7°, 180° scan, and 3 frame averaging. CT scans were reconstructed using NRecon software (Micro Photonics, Allentown, PA) and rendered in Drishti Volume Exploration and Presentation Tool [38] (Open Access, Australian Capital Territory) with consistent editing parameters. Images were processed to isolate the skull cap from the rest of the skull to improve suture visualization in 3D. Phenotyping was conducted blindly as to genotype and scored as unaffected or unilateral or bilateral fusion of the coronal suture. Though metopic, coronal, sagittal, and lambdoid sutures were all analyzed, craniosynostosis was only observed in the coronal sutures. In this study, we considered bilateral synostosis to be a more severe phenotype than unilateral synostosis. We also observed a subtype of fusion we call “bridging,” defined as a small segment of fusion resembling a bridge across the otherwise patent suture. Although we do not take this into consideration with regard to severity of the disease in our mice, we make note of this phenotype in S1 Fig.

### Statistical analysis

#### Expression analysis

A paired t-test assuming equal variance was performed to compare the expression levels of each gene between the 23 highest IGF1 expressing cases and both the remaining 188 cases and the 50 unaffected controls.

#### Mouse phenotype proportion analysis

Statistical analysis was performed using a chi-squared test for comparison of proportions. Percent of expected affected for each compound heterozygous genotype was calculated by the sum of observed affected in each component genotype. Proportions were then compared using an n of 17 for the comparison of *Igf1*^(+/tg)^ and *Twist1*^(+/-)^ to *Twist1*^(+/-)^/*Igf1*^(+/tg)^ and an n of 18 for the comparison of *Gsk3β*^(+/-)^ and *Twist1*^(+/-)^ to *Twist1*^(+/-)^/*Gsk3β*^(+/-)^.

## Results

### cDNA microarray and expression analysis

**Fig 1:**
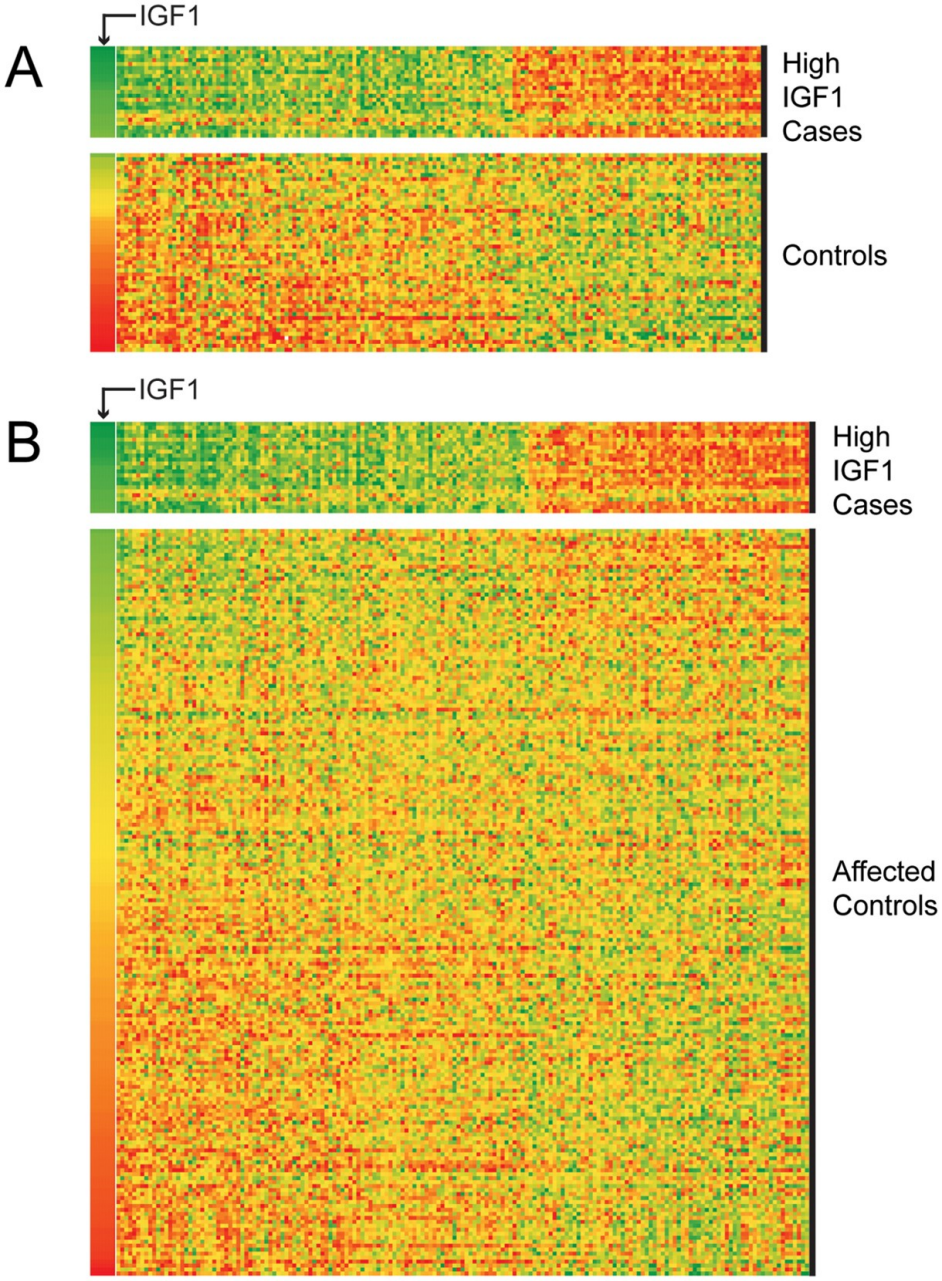
Heat map representation of RNA expression of genes involved with bone biology. The candidate genes of interest depicted in this heat map have a statistically significant difference in expression between (A) 23 cases with the highest IGF1 expression levels and unaffected controls and (B) 23 cases with the highest IGF1 expression levels and 188 remaining cases (affected controls). Affected controls represent SSC cases with lower IGF1 expression. IGF1 expression is shown in the far-left column (arrow) and sorted high to low. The expression of each transcript is represented with conditional formatting with green indicating highest and red representing lowest expression among individuals. Candidate gene expression is ordered left to right by the ratio of the mean expression value of the top 23 (high IGF1 cases) to the mean of unaffected controls (A) and affected control cases (B). See S1 Table for the complete gene list and statistical significance.

Our initial data review identified a striking association between IGF1 expression and many of the 399 candidate genes chosen a priori due to their role in osteogenesis and bone development. Comparison of the expression levels of our candidate genes in the 23 cases with the highest levels of IGF1 expression (High IGF1 Cases) and both the 50 unaffected controls (Fig 1A) and the remaining 188 cases (affected controls; Fig 1B) revealed strong correlations between IGF1 expression and many of our candidate genes. The expression of 161 transcripts was significantly different between the High IGF1 Case subset and unaffected controls and the expression of 173 transcripts was significantly different between the High IGF1 Case subset and the affected controls. These data reveal that cases with increased IGF1 expression demonstrate a pattern of osteoblast gene expression not found in control osteoblasts or cases with lower IGF1 expression (S1 Table). Notably, we identified that expression of *RUNX2*, a transcription factor critical for osteogenesis, was correlated with IGF1 expression and was significantly different between High IGF1 cases and unaffected controls (p-value = 8.61x10-4) as well as affected controls (p-value = 1.42x10-5).

### MicroCT phenotyping

We analyzed 3D-rendered CT images of congenic, postnatal day 28 (P28) C57BL/6J mice with the following single-variant genotypes; *Gsk3β*^(+/-)^, *Twist1*^(+/-)^, *Igf1*^(+/tg)^, *Igf1*^(tg/tg)^, and with the following compound- heterozygous variant genotypes; *Gsk3β*^(+/-)^/*Twist1*^(+/-)^, *Twist1*^(+/-)^/*Igf1*^(+/tg)^, and *Gsk3β*^(+/-)^/*Igf1*^(+/tg)^ (Table 2, S1 Fig). Due to technical factors or postnatal death, 9 mice were scanned at days other than P28 (see S1 Fig). We observed no instances of suture fusion in *Igf1*^(+/tg)^, *Igf1*^(tg/tg)^, or wildtype mice. Fifteen percent of *Gsk3β*^(+/-)^ mice had suture fusion, one of which had increased biparietal diameter suggestive of hydrocephalus. One *Gsk3β*^(+/-)^ mouse without suture fusion also had increased biparietal diameter. Based on the suture morphology we suspect that this mouse had bilateral coronal suture fusion that was disrupted due to pressure from brain growth and concomitant hydrocephalus. This animal was classified as not having craniosynostosis (S1 Fig). Thirty-five percent of *Twist1* ^(+/-)^ mice had suture fusion.

**Table 2:** Phenotypes of each mouse strain and heterozygous cross.

				Of Total	Of Total	Of Affected	Of Affected
Genotype	Total n	Normal	Affected	Unilateral	Bilateral	Unilateral	Bilateral
**C57BL/6J**	7	7 (100%)	-	-	-	-	-
***Igf1*^(+/tg)^**	7	7 (100%)	-	-	-	-	-
***Igf1*^(tg/tg)^**	5	5 (100%)	-	-	-	-	-
***Gsk3β*^(+/-)^**	13	11 (84.6%)	2 (15.4%)	1 (7.7%)	1 (7.7%)	1 (50%)	1 (50%)
***Twist1*^(+/-)^**	17	11 (64.7%)	6 (35.3%)	5 (29.4%)	1 (5.9%)	5 (83.3%)	1 (16.7%)
***Gsk3β*^(+/-)^/*Igf1*^(+/tg)^**	7	7 (100%)	-	-	-	-	-
***Twist1*^(+/-)^/*Gsk3β*^(+/-)^**	18	1 (5.6%)	17 (94.4%)	14 (77.8%)	3 (16.7%)	14 (82.4%)	3 (17.6%)
***Twist1*^(+/-)^ / *Igf1*^(+/tg)^**	17	9 (52.9%)	8 (47.1%)	3 (17.6%)	5 (29.4%)	3 (37.5%)	5 (62.5%)

Summary of craniosynostosis frequency and sub-phenotype (unilateral and bilateral) in mice with single and compound genotypes of interest

**Fig 2:**
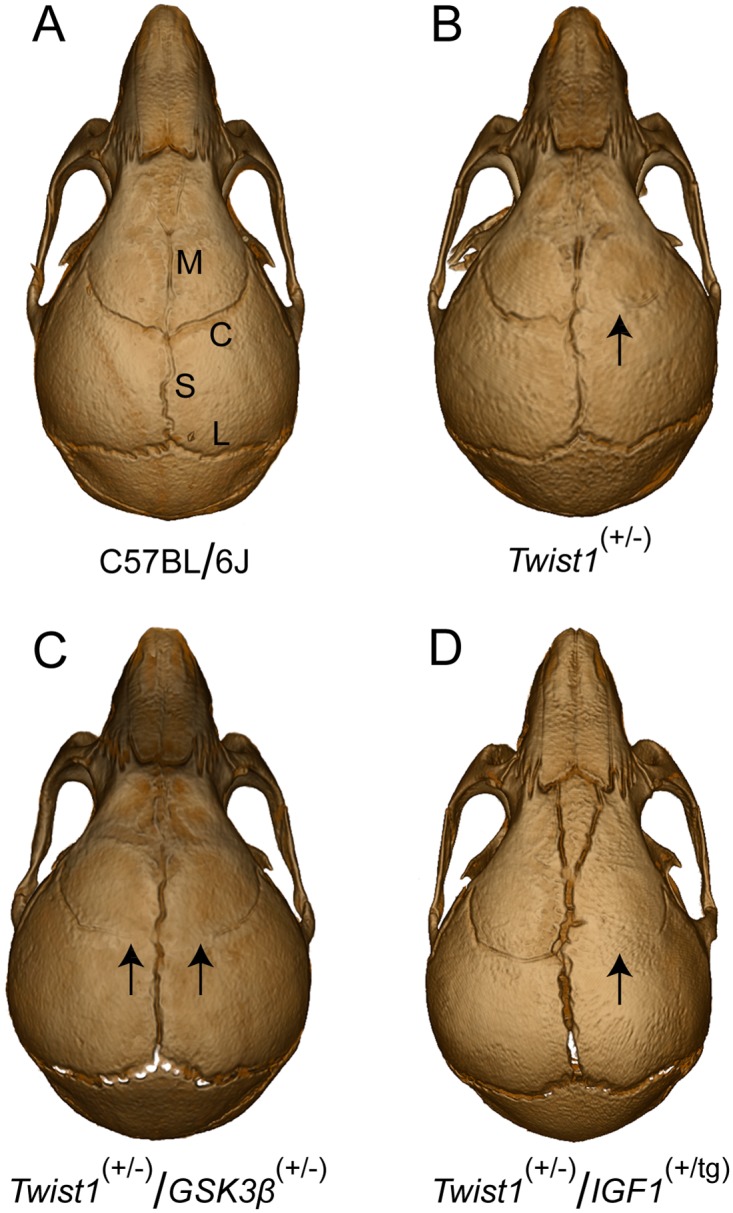
MicroCT images of representative P28 skulls. (A) wildtype C57BL/6J mouse with normal calvarial suture pattern, (B) *Twist1*^(+/-)^ mouse with right coronal craniosynostosis, (C) *Twist1* ^(+/-)^/*Gsk3β*^(+/-)^ mouse with partial bilateral coronal craniosynostosis, and (D) *Twist1*^(+/-)^/*Igf1*^(+/tg)^ mouse with right coronal craniosynostosis. Sutures are labelled as follows: M (metopic or interfrontal), C (coronal), S (sagittal), and L (lambdoid). Arrows designate areas of premature suture fusion.

**Fig 3:**
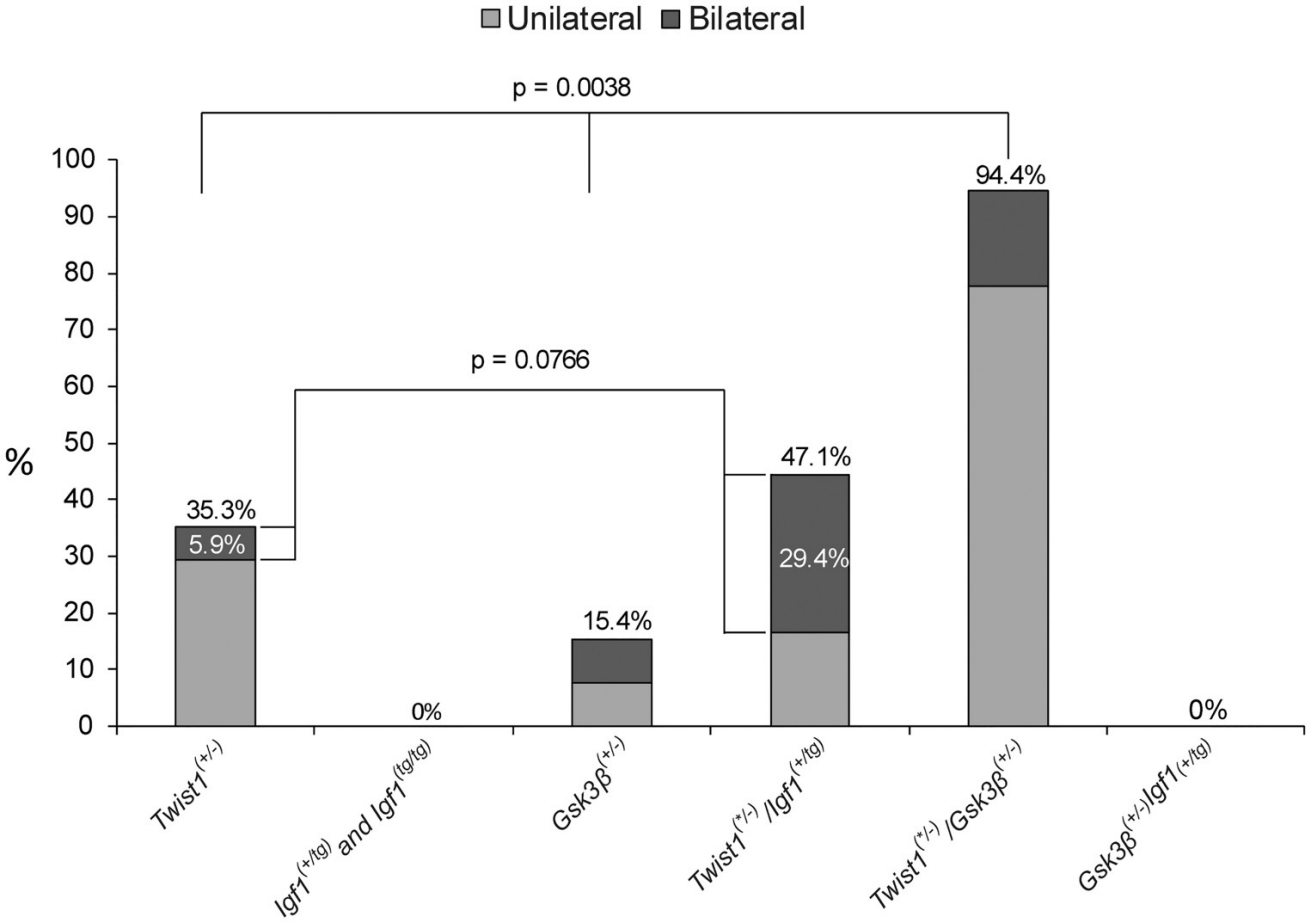
Percentage of mice with craniosynostosis by genotype. Mice that harbored mutations in both *Twist1* ^(+/-)^ and *Gsk3β*^(+/-)^ showed a significant increase in rate of craniosynostosis as compared to the expected additive outcome of *Twist1* ^(+/-)^ and *Gsk3β*^(+/-)^ independently (based on their individual rates of craniosynostosis). Mice that harbored mutations in both *Twist1* ^(+/-)^ and *Igf1*^(+/tg)^ showed a near-significant increase in instances of bilateral craniosynostosis as compared to the expected additive outcome of bilateral craniosynostosis in *Twist1* ^(+/-)^ and *Igf1*^(+/tg)^ (or *Igf1*^(tg/tg)^) independently.

The instance of suture fusion increased from 35% in *Twist1* ^(+/-)^ and 15% in *Gsk3β*^(+/-)^ to 94% in *Twist1*^(+/-)^/*Gsk3β*^(+/-)^, a significance of p = 0.0038 (accounting for the additive effect of these disease-causing genotypes). Of note, inclusion of the mouse with suspected craniosynostosis in our analysis did not change the interpretation of our results (p = 0.0121). There was no significant increase in the severity of craniosynostosis when comparing these groups. The incidence of fusion was not significantly increased in *Twist1*^(+/-)^/*Igf1*^(+/tg)^ as compared to *Twist1* ^(+/-)^ mice, but the severity (bilateral) increased from 6% of *Twist1* ^(+/-)^ to 29% of *Twist1*^(+/-)^/*Igf1*^(+/tg)^ (p = 0.0766) (Figs 2 and 3).

We observed a disproportionally lower number of mice with the *Gsk3β*^(+/-)^/*Igf1*^(+/tg)^ genotype than predicted by Hardy-Weinberg equilibrium (~10% as compared to the expected 25%), suggesting the possibility of prenatal lethality of this genotype. While none of the *Gsk3β*^(+/-)^/*Igf1*^(+/tg)^ mice that survived to P28 showed suture fusion, the reduced number of offspring with this genotype precludes our ability to define the true craniosynostosis rate.

## Discussion

While there have been great advances in our understanding of the molecular genetic causes of syndromic craniosynostosis, the etiology of most cases of the more common single suture synostoses remains unknown. Its low recurrence rate suggests multifactorial, polygenic, or oligogenic inheritance. In this study, we describe compound heterozygous mutant mice with genetically altered expression of *Igf1* and *Gsk3β* and disinhibition of *Runx2*, that result in a significantly increased rate or severity of craniosynostosis when compared to mice with each genotype in isolation.

**Fig 4:**
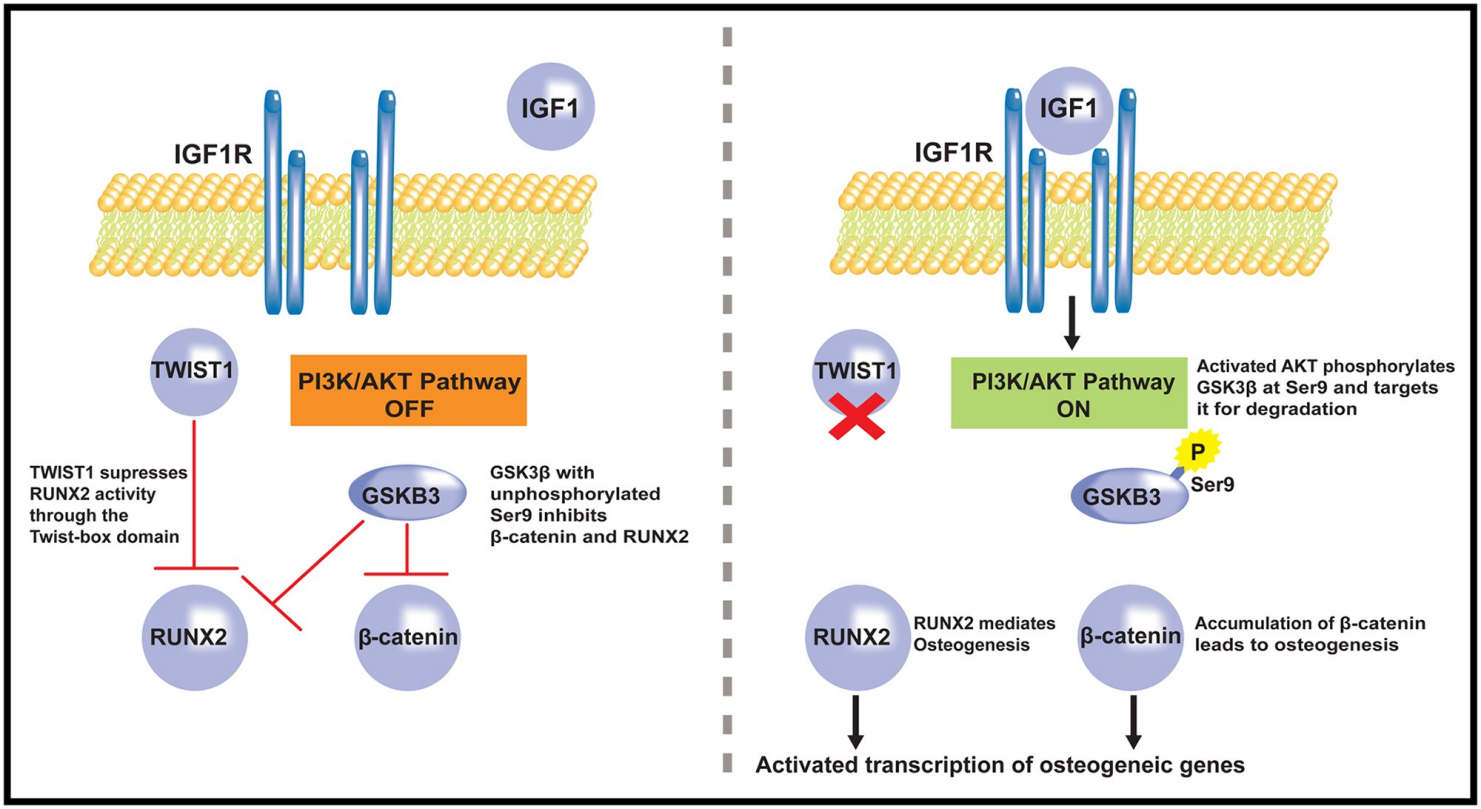
Schematic representation of intersection of pathways of interest. (Left Panel) In the absence of IGF1/IGF1R binding, the Akt pathway is inactive and GSK3β Ser9 is unphosphorylated resulting in inhibition of RUNX2 (through phosphorylation of S369, S373, S377) and β-catenin. In parallel, TWIST1 suppresses RUNX2 function via direct interaction of RUNX2 with the twist-box domain of TWIST1. (Right Panel) In our experimental system we tested the hypothesis that dysregulation of both the TWIST1/RUNX2 and IGF1/Akt pathways would result in an increased incidence of craniosynostosis. This schematic demonstrates our hypothesis that the combination of a twist-box mutation of *Twist1* and activation of the IGF1/AKT pathway would result in activation of osteogenic transcripts and manifest as craniosynostosis. The pathways shown are derived from previously described data [19, 41, 43, 44].

Our mouse models were based on human expression data demonstrating a positive correlation between *IGF1* and *RUNX2* expression in a subgroup of osteoblasts derived from clinical cases of SSC. The IGF1/IGF1R pathway is critical for normal bone growth and density [39] and has been implicated in the etiology of craniosynostosis [9, 10]. IGF1 signaling through IGF1R activates the Akt pathway, inhibits GSK3β, and results in disinhibition of β-catenin and RUNX2, promoting osteogenesis [40–42]. GSK3β is known to attenuate RUNX2 transcriptional activity though phosphorylation (Ser369, Ser373, Ser377) and Twist1 inhibits RUNX2 function through protein-protein intearction at the twist-box. Thus, our mouse models *Igf1*^*(+/tg)*,^ *Twist1*^(+/-)^, and *Gsk3β*^(+/-)^ converge to reduce inhibition and increase the transcriptional activity of Runx2. The mouse models selected for our work mimicked activation of the Akt pathway and selective disinhibition of RUNX2 to determine the additive effects of pathway dysregulation (Fig 4.) [18, 41, 43, 44].

The *Twist1*^(+/-)^ mouse is known to harbor a point mutation in the twist-box resulting in selective disinhibition of RUNX2 leading to coronal synostosis of variable penetrance and expressivity. This was validated in our study demonstrating that 35% of *Twist1*^(+/-)^ mice developed coronal synostosis.

While none of the *Igf1*^(+/tg)^ or *Igf1*^(tg/tg)^ transgenic mice developed craniosynostosis, 23% of the *Gsk3β*^(+/-)^ mice displayed craniosynostosis and/or presumed hydrocephalus, a previously undescribed phenotype with incomplete penetrance. With this unexpected result, we reviewed recent sequence data from our cohort and identified a missense mutation (c.T185C:p.I62T) in a single case of sagittal synostosis. This variant has not been identified in more than 250,000 alleles sequenced [45, 46], resides in both an ATP binding domain and the catalytic domain of the Serine/Threonine Kinase, and is conserved in all vertebrates including zebrafish. This finding not only supports the role of GSK3β in polygenic inheritance but suggests that isolated missense mutations may cause craniosynostosis. An additional trend that we observed is an exaggerated bifurcation of the interfrontal suture in *Twist1*^(+/-)^/*Gsk3β*^(+/-)^ compound heterozygous mice. While is is known that C57B/6J mice naturally present with an “interfrontal bone” [47] and thus bifurcated interfrontal sutures, we see that our compound heterozygotes have noticeably more defined and obtuse bifurcation. The significance of this observation is unknown.

Compound heterozygous *Twist1*^(+/-)^/*Gsk3β*^(+/-)^ had a significantly higher frequency of craniosynostosis than predicted by the additive effects of each mutation alone (increasing from 35% in *Twist1*^(+/-)^ and 15% in *Gsk3β*^(+/-)^ to 94% in *Twist1*^(+/-)^/*Gsk3β*^(+/-)^ compound heterozygotes). Furthermore, there was a near significant increase in the rate of bilateral coronal synostosis in *Twist1*^(+/-)^/*Igf1*^(+/tg)^ compound heterozygotes when compared to the rate in the parental strains.

These data suggest that a functional interaction between the TWIST1/RUNX2 and IGF1 pathways is integral to normal calvarial development. We have provided data supporting that combined dysregulation of these pathways result in the first example of polygenic inheritance of craniosynostosis in an animal model. While our data suggest that dysregulation of the TWIST1/RUNX2 and IGF1/Akt pathways may predispose humans to craniosynostosis, further translational research is necessary before the development of clinical utility.

## Supporting information

S1 Fig MicroCT images of all mice included in this study. The phenotype of 91 mice was classified in this study. Each skull image is designated by genotype and pattern of suture fusion (U) Unilateral craniosynostosis, (Ub) Unilateral craniosynostosis with “bridging” phenotype, (B) Bilateral craniosynostosis, or (Bb) Bilateral craniosynostosis with “bridging” phenotype. (*) Irregular and wide suture margins and wide biparietal diameter of this mouse suggests the possibility bilateral coronal suture fusion that was disrupted due to concomitant hydrocephalus. This animal was classified as not having craniosynostosis. (**) Mouse died at P23 with potential hydrocephalus based on increased bi-parietal diameter. Postnatal age at the time of sacrifice is noted if other than P28.
(PDF)

S1 Table Genes with significantly altered expression in cases with high IGF1 expression. Alpha list of 399 genes of interest chosen a priori for their role in osteoblast development and bone biology. (§) indicates significant difference in expression levels between 23 cases with the highest levels of *IGF1* expression and 50 unaffected controls. (¥) indicates significant difference in expression levels between 23 cases with the highest levels of *IGF1* expression and 188 affected control cases (e.g. cases with craniosynostosis with low *IGF1* expression). (↑) indicates a the gene is positively correlated with *IGF1* and (↓) indicates that a gene is inversely correlated with *IGF1* for the group(s) it is significant in.
(PDF)

S2 Table Official mouse strain names and abbreviations used in manuscript. (PDF)

S3 Table Primers used to genotype each mouse strain. Primer sequences for *Igf1 and Gsk3β* sourced from Jackson Laboratories genotyping protocols (jax.org). Primers spanning *Twist1* Ser192Pro designed in-house.
(PDF)
