# Supplementary material for: Calvarial osteoblast gene expression in patients with craniosynostosis leads to novel polygenic mouse model
Source: PLoS One. 2019 Aug 23;14(8):e0221402. doi: 10.1371/journal.pone.0221402 (PMC6707563; doi:10.1371/journal.pone.0221402)
Supplement: S1 Fig — The phenotype of 91 mice was classified in this study. Each skull image is designated by genotype and pattern of suture fusion (U) Unilateral craniosynostosis, (Ub) Unilateral craniosynostosis with “bridging” phenotype, (B) Bilateral craniosynostosis, or (Bb) Bilateral craniosynostosis with “bridging” phenotype. (*) Irregular and wide suture margins and wide biparietal diameter of this mouse suggests the possibility bilateral coronal suture fusion that was disrupted due to concomitant hydrocephalus. This animal was classified as not having craniosynostosis. (**) Mouse died at P23 with potential hydrocephalus based on increased bi-parietal diameter. Postnatal age at the time of sacrifice is noted if other than P28. (PDF) [file pone.0221402.s001.pdf]

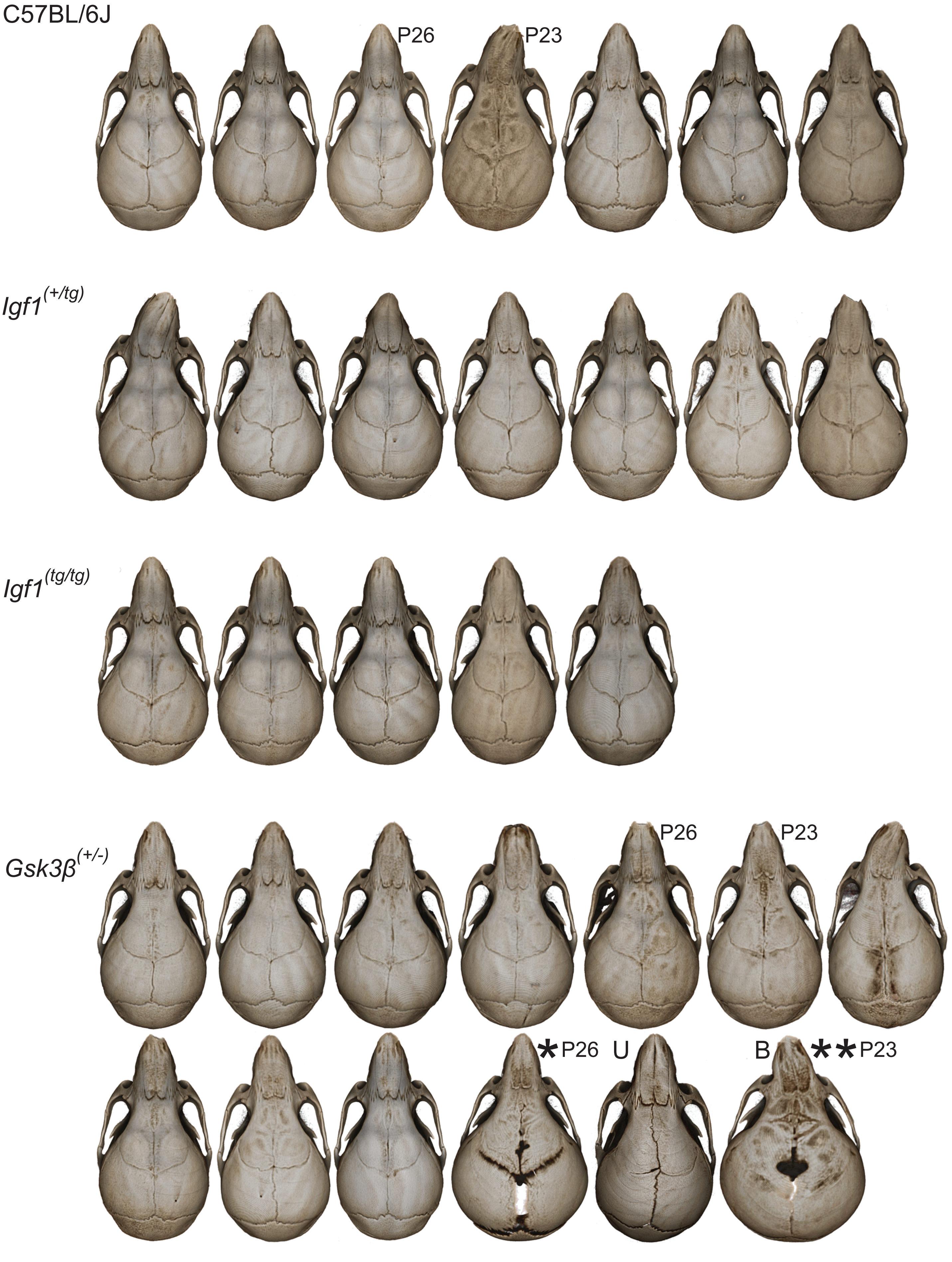

C57BL/6J
P26
P23
Igf1(+/tg)
Igf1(tg/tg)
Gsk3β(+/-)
P26
P23
* P26
U
B
** P23

Twist1$^{(+/-)}$

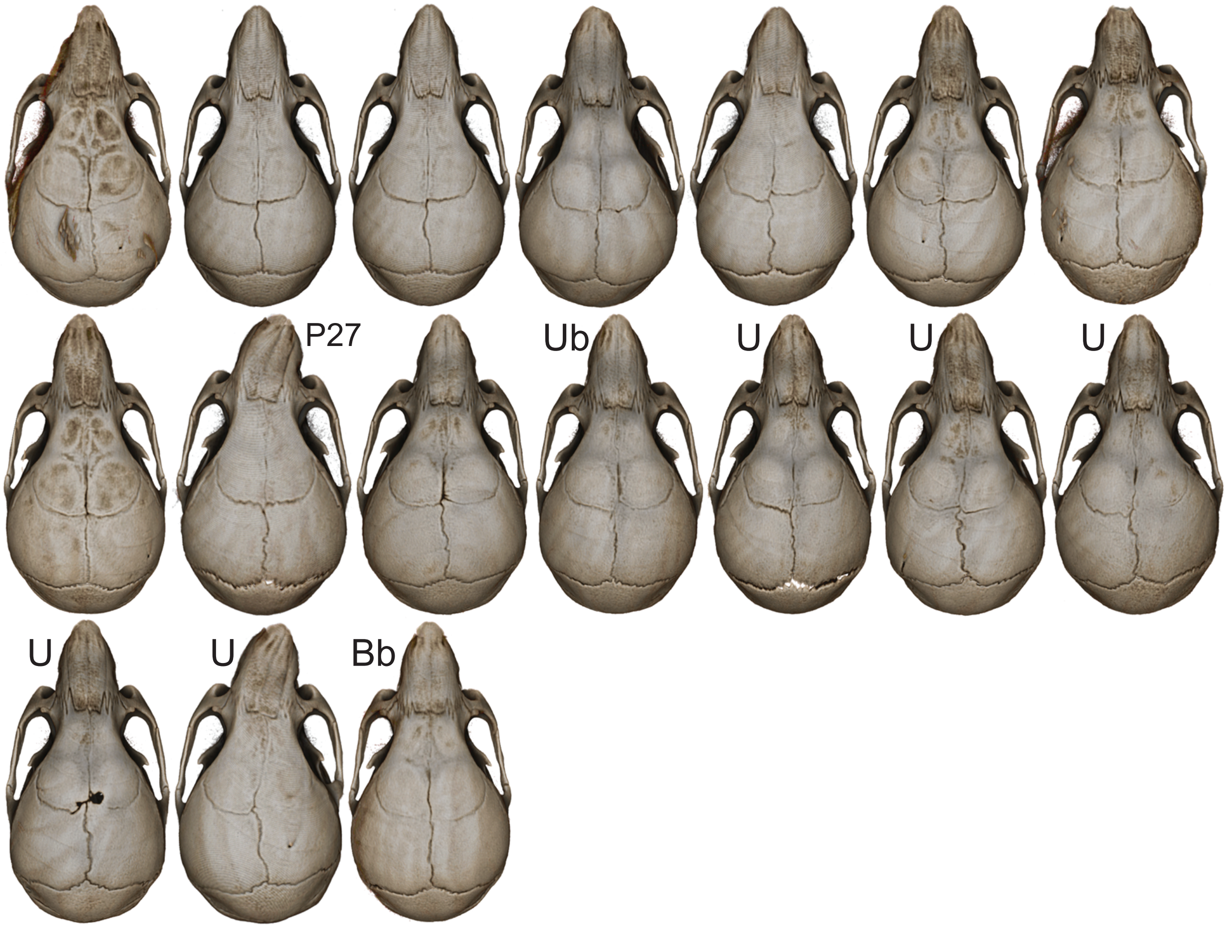

Twist1$^{(+/-)}$/*Gsk3β*$^{(+/-)}$

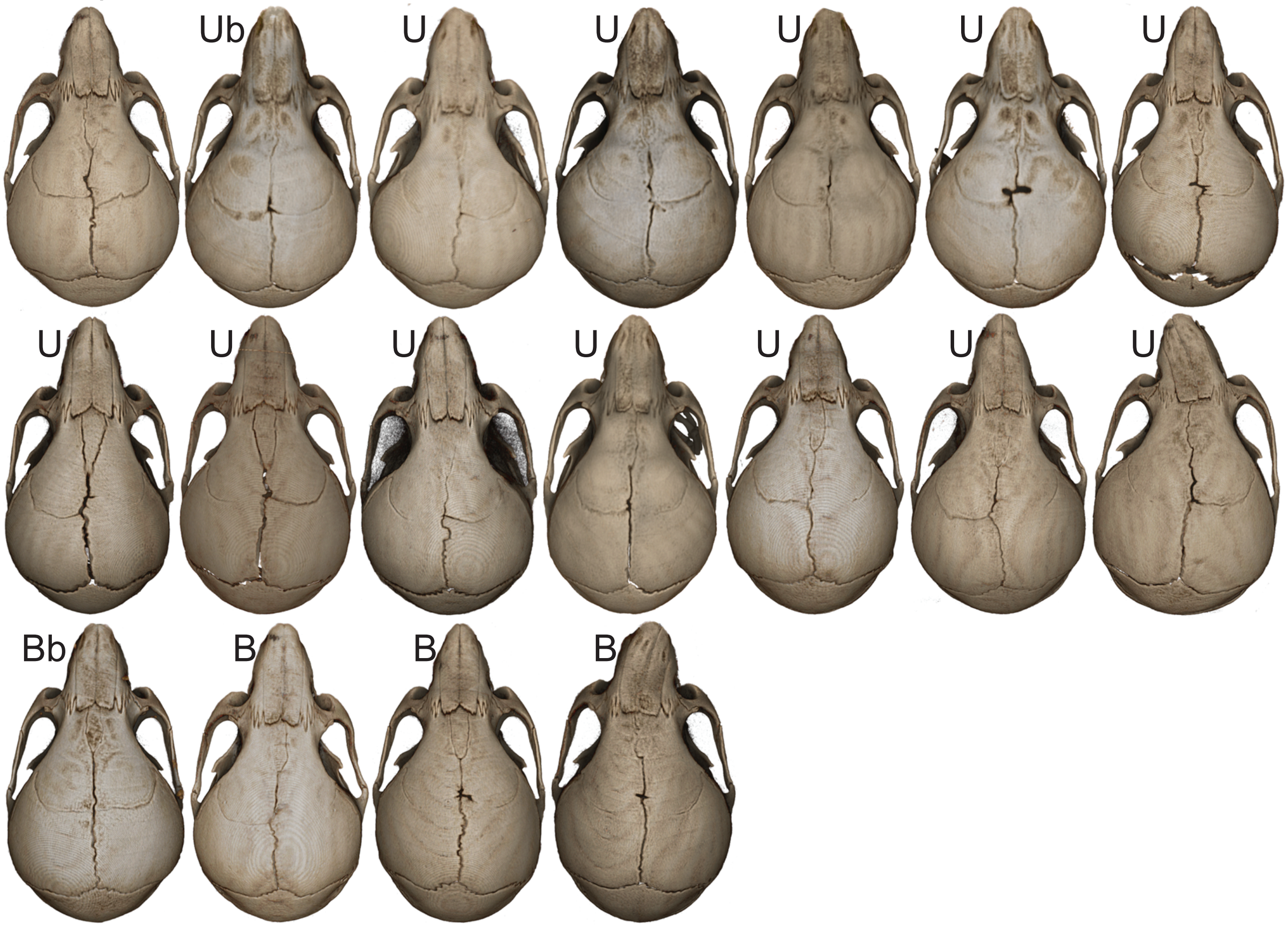

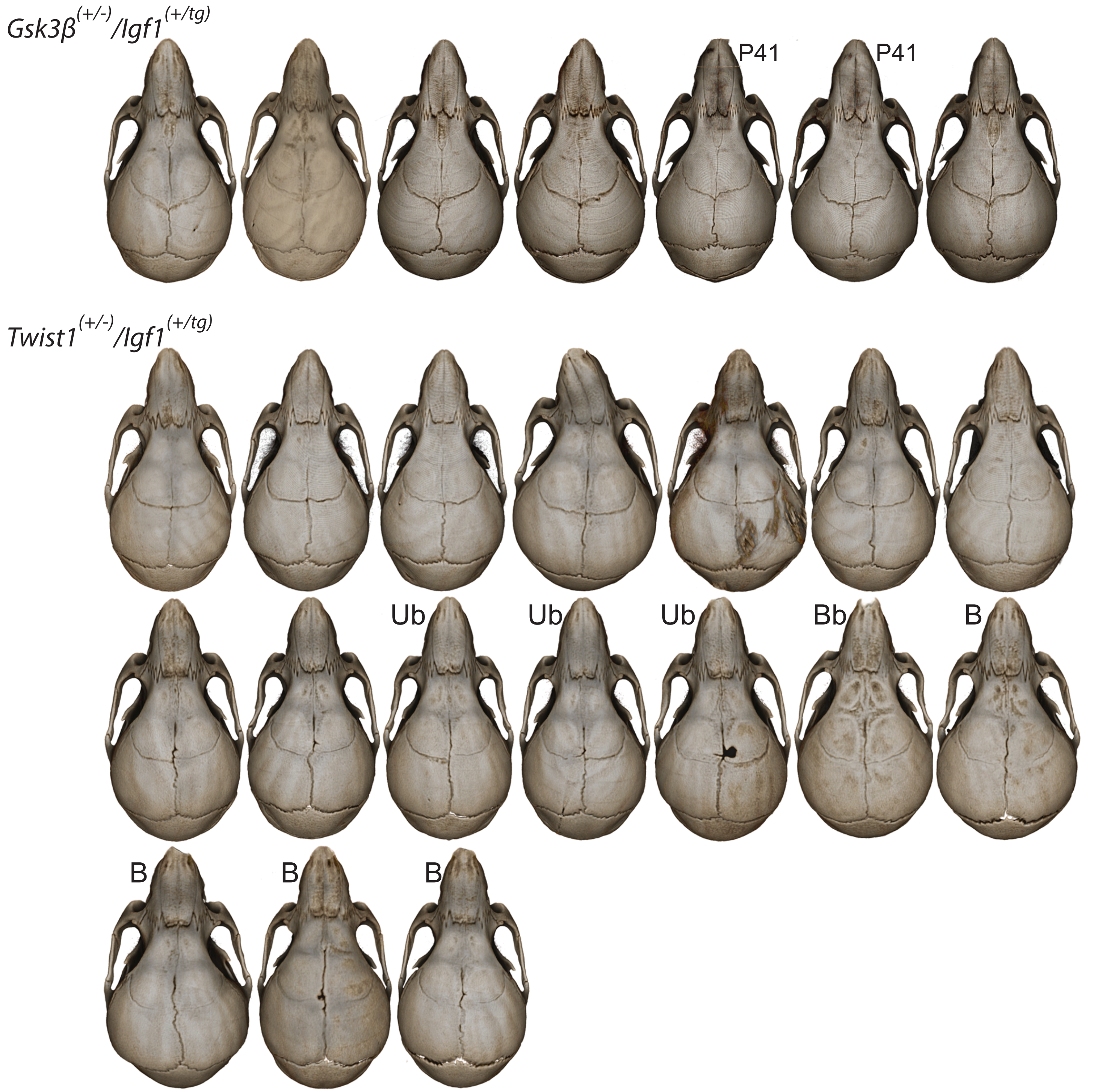

Supplemental Figure 1. MicroCT images of all mice included in this study.

The phenotype of 91 mice was classified in this study. Each skull image is designated by genotype and pattern of suture fusion: (U) Unilateral craniosynostosis, (Ub) Unilateral craniosynostosis with “bridging” phenotype, (B) Bilateral craniosynostosis, or (Bb) Bilateral craniosynostosis with “bridging” phenotype. (*) Irregular and wide suture margins and wide biparietal diameter of this mouse suggests the possibility bilateral coronal suture fusion that was disrupted due to concomitant hydrocephalus. This animal was classified as not having craniosynostosis. (**) Mouse died at P23 with bilateral coronal synostosis and potential hydrocephalus based on increased bi-parietal diameter. The postnatal age at the time of sacrifice is noted if other than P28.
