# Supplementary material for: Calvarial osteoblast gene expression in patients with craniosynostosis leads to novel polygenic mouse model
Source: PLoS One. 2019 Aug 23;14(8):e0221402. doi: 10.1371/journal.pone.0221402 (PMC6707563; doi:10.1371/journal.pone.0221402)
Supplement: S1 Table — Alpha list of 399 genes of interest chosen a priori for their role in osteoblast development and bone biology. (§) indicates significant difference in expression levels between 23 cases with the highest levels of IGF1 expression and 50 unaffected controls. (¥) indicates significant difference in expression levels between 23 cases with the highest levels of IGF1 expression and 188 affected control cases (e.g. cases with craniosynostosis with low IGF1 expression). (↑) indicates a the gene is positively correlated with IGF1 and (↓) indicates that a gene is inversely correlated with IGF1 for the group(s) it is significant in. (PDF) [file pone.0221402.s002.pdf]

Supplemental Table 1. Alphabetical list of genes analyzed from cDNA microarray for this study

Gene Name		Gene Name		Gene Name		Gene Name		Gene Name		Gene Name		Gene Name		Gene Name	
ACAN		CDH6		EFNA4		FGF9	¥§↑	ID4	¥§↑	KRT15	¥§↓	RARA	¥§↑	TGFB3	¥§↑
ACTG2		CDKN1A		EFNA5	¥§↑	FGFBP1		IGF1	¥§↑	LAMA1		RARB	¥§↓	TGFBI	¥§↓
ACTN4	¥§↓	CDKN1B		EFNB1		FGFBP2		IGF1R	¥↑	LAMA2	¥§↑	RARG	¥↑	TGFBR1	¥§↑
ACVR1	¥§↑	CDKN1C	§↑	EFNB2		FGFBP3		IGF2	¥§↑	LAMA4	¥§↑	RARRES1		TGFBR2	
ACVRL1	¥↓	CDKN2A		EFNB3		FGFR1		IGF2AS		LAMA5		RARRES2	¥§↑	TGFBR3	
AEBP1		CDKN2B		EGF		FGFR1OP		IGF2BP1	¥↓	LAMC2	¥§↓	RARRES3	¥§↑	TGFBRAP1	¥§↑
AFF2	¥§↑	CDKN2C		EGFR	¥§↑	FGFR1OP2		IGF2BP2	§↑	LEF1	¥§↓	REL		TGIF1	¥§↑
AHSG		CDKN2D		EGR3		FGFR2	¥§↑	IGF2BP3	¥↓	LEP		RELA	§↓	TGIF2	
ALDH1A3		CDKN3	¥§↓	ENAM		FGFR3	¥↑	IGF2R	¥§↑	LEPR	¥§↑	RELB	¥§↓	TGIF2LX	
ALPL		CHI3L1		ENPP2	¥§↑	FGFR4		IGFALS		LGALS3		RGS12		TGIF2LY	
ALX1	¥↓	cldn1	§↑	EPHA1		FGFRL1	¥§↑	IGFBP1		LGALS3BP		RUNX1	§↑	THBS2	¥§↑
AMBN		CNTNAP3		EPHA10		FGL2	¥§↑	IGFBP2		LOXL4	¥§↓	RUNX1T1	¥§↑	THRA	¥§↑
AMELY		COL10A1		EPHA2	¥§↓	FLNC	¥§↓	IGFBP3		LPPR4		RUNX2	¥§↑	THRAP3	
ANXA5		COL11A1	¥§↑	EPHA3	¥§↑	FLT1		IGFBP4	¥§↑	LRP1	¥§↑	RUNX3		THRB	¥§↑
BGLAP	¥§↓	COL12A1	¥§↑	EPHA4	¥§↑	FN1	¥§↓	IGFBP5	¥§↑	LUZP2	¥§↑	S100A4		THRSP	
BGN		COL14A1	¥§↑	EPHA5		FOXC1	¥§↑	IGFBP6	¥§↓	MCAM		SCARB1		TINAGL1	¥§↓
BMP1		COL15A1		EPHA6		FOXE1		IGFBP7		MINPP1	¥§↓	SDC4	¥§↓	TLR4	
BMP10		COL1A1	§↑	EPHA7		FOXF1		IGFBPL1		MME	¥§↑	SEMA3C	§↑	TNF	
BMP15		COL1A2	¥§↑	EPHA8		FOXN3		INSR		MMP10		SEMA3D	¥§↑	TNFRSF11B	
BMP2		COL2A1		EPHB1	¥§↓	FOXO1	§↑	IRS1	¥↑	MMP2		SERPINH1	¥§↓	TOX	¥§↑
BMP2K		COL3A1	¥§↑	EPHB2		FOXO3		IRS2	¥↑	MMP8		SFRP1	¥↑	TUBB3	¥§↓
BMP3		COL4A1		EPHB3		FOXO3B		IRS4		MMP9		SFRP2	¥↑	TUFT1	
BMP4		COL4A2		EPHB4		FOXO4	¥§↓	ITGA10	¥§↓	MSX1	¥§↑	SFRP4	§↑	TWIST1	¥↑
BMP5		COL4A3		EPHB6		FZD1	¥§↑	ITGA11	§↑	MSX2	¥§↑	SFRP5		VCAM1	§↓
BMP6	§↑	COL5A1		FAM38B	¥§↑	FZD10		ITGA2	¥§↓	MYOCD		SHC4		VDR	
BMP7		COL5A2		FBN1	¥§↑	FZD2		ITGA2B		NCAM1		SLC7A2	¥§↑	VEGFA	¥↑
BMP8A		COL6A3		FBN2	¥§↑	FZD3	¥§↑	ITGA3	¥§↓	NEDD9	¥§↓	SMAD1		VEGFB	¥§↑
BMP8B		COMP		FBN3		FZD4		ITGA4	¥§↓	NEFM	¥§↓	SMAD2	¥§↑	WFS12	
BMPER	¥§↑	CSF2		FBXO32	¥↑	FZD5	¥§↑	ITGA5	¥§↓	NELL1	¥§↓	SMAD3	¥§↓	WNT1	
BMPR1A	¥§↑	CSF3		FGF1	¥§↓	FZD6		ITGA6		NELL2		SMAD4	¥§↑	WNT10A	
BMPR1B	¥§↑	CTNNB1	¥§↑	FGF10	¥§↑	FZD7		ITGA7		NFIX	¥↑	SMAD5		WNT10B	
BMPR2	¥§↑	CTSK	¥§↑	FGF11		FZD8	¥§↑	ITGA8		NFKB1		SMAD6	¥§↑	WNT11	
CALCR		CXCL12		FGF12		FZD9		ITGA9		NFKB2	¥§↓	SMAD7	¥§↑	WNT16	¥§↓
CALCRL	¥§↓	DKK1	¥§↓	FGF13		GCNT4		ITGAD		NLRP1		SMAD9	¥§↑	WNT2	¥↓
CAV2	¥§↓	DKK2	¥§↑	FGF14	¥§↑	GDF10		ITGAE		OGN	¥§↑	SNAI1		WNT2B	¥§↑
CCNA1		DKK3		FGF16	¥§↓	GFRA1	¥↑	ITGAL	¥↓	OXTR	¥§↓	SNAI2		WNT3	
CCNA2	¥§↓	DKK4		FGF17		GH1		ITGAM		pappa	¥§↑	SNAI3		WNT3A	
CCNB1	¥§↓	DLL1		FGF18	¥§↑	GH2		ITGAV	¥§↓	pappa2	§↑	SOX9		WNT4	
CCNB2	¥§↓	DLX1	¥§↓	FGF19		GHDC	¥§↑	ITGAX		PARD6A		SP7		WNT5A	
CCNB3		DLX2	§↓	FGF2		GHITM		ITGB1	¥§↓	PDE1A	¥§↑	SPARC		WNT5B	¥§↓
CCNC	¥§↓	DLX3		FGF20		GHR	¥§↑	ITGB2		PDE1B		SPP1		WNT6	
CCND1	¥§↓	DLX4		FGF21	¥§↓	GHRH		ITGB3	¥§↓	PDE1C	¥↑	STATH		WNT7A	
CCND2	¥§↑	DLX5	¥§↑	FGF22		GHRHR		ITGB3BP		PDE8B	¥§↑	SULT1E1		WNT7B	
CCND3	¥§↓	DLX6	¥§↑	FGF23		GHRL		ITGB4		PDGFA		TBX18	¥§↑	WNT8A	¥↓
CCNE1	¥§↓	DMP1		FGF3		GLI3	¥§↑	ITGB5	¥↑	PDGFC	¥↓	TCF7	¥§↓	WNT8B	
CCNE2	¥§↓	DSG2		FGF4		GLIS3	¥§↑	ITGB6		PENK	¥§↑	TFIP11		WNT9A	
CD36		DSPP		FGF5	¥§↓	GREM2	¥↑	ITGB7		PHEX	¥§↓	TGFA		WNT9B	
CD44	¥§↓	EFNA1		FGF6		GSK3B	¥§↑	ITGB8	¥↑	PLA2G5	¥↓	TGFB1	¥§↓	WWTR1	
CDC2	¥§↓	EFNA2		FGF7	¥§↑	IBSP		ITGBL1	¥§↑	PRRX2	¥↑	TGFB1I1	¥§↓	ZFHX4	¥§↑
CDH11		EFNA3		FGF8		ICAM1	¥§↓	JAG1		PTTG1IP		TGFB2			

Alphabetical list of 399 genes of interest chosen a priori for their role in osteoblast development and bone biology. (§) indicates significant difference in expression levels between 23 cases with the highest levels of *IGF1* expression and 50 unaffected controls. (¥) indicates significant difference in expression levels between 23 cases with the highest levels of *IGF1* expression and 188 affected control cases (e.g. cases with craniosynostosis with low *IGF1* expression). (↑) indicates a the gene is positively correlated with *IGF1* and (↓) indicates that a gene is inversely correlated with *IGF1* for the group(s) it is significant in.
