# Supplementary material for: Calvarial osteoblast gene expression in patients with craniosynostosis leads to novel polygenic mouse model
Source: PLoS One. 2019 Aug 23;14(8):e0221402. doi: 10.1371/journal.pone.0221402 (PMC6707563; doi:10.1371/journal.pone.0221402)
Supplement: S2 Table — (PDF) [file pone.0221402.s003.pdf]

S2 Table: Official mouse strain names and abbreviations used in manuscript

Jackson Laboratories Official Strain Name	Abbreviation for Manuscript
FVB-Tg(Ttr-Igf1)1Sykr/J	*Igf1*$^{(+/tg)}$, *Igf1*$^{(tg/tg)}$
B6.129P2-*Gsk3b*^{tm1Dgen}/J	*Gsk3β*$^{(+/-)}$
C3;B6-*Twist1*^{ska10}/Mmcd	*Twist1*$^{(+/-)}$
