# Supplementary material for: Calvarial osteoblast gene expression in patients with craniosynostosis leads to novel polygenic mouse model
Source: PLoS One. 2019 Aug 23;14(8):e0221402. doi: 10.1371/journal.pone.0221402 (PMC6707563; doi:10.1371/journal.pone.0221402)
Supplement: S3 Table — Primer sequences for Igf1 and Gsk3β sourced from Jackson Laboratories genotyping protocols (jax.org). Primers spanning Twist1 Ser192Pro designed in-house. (PDF) [file pone.0221402.s004.pdf]

Supplemental Table 3. Primers used to genotype each mouse strain.

Amplicon Gene	Primer Name	Primer Sequence
**Twist1**	Twist1-F	5’-ACGCAGTCGCTGAACGAGG-3’
	Twist1-R	5’-AGGAGAGTGGAGGGGGATGG-3’
**Gsk3b**	Gsk3b-1	5’-GGGTGGGATTAGATAAATGCCTGCTCT-3’
	Gsk3b-2	5’-AGGGATATGGTTCGGTAGTTAAGAG-3’
	Gsk3b-3	5’-GCAAGGTAACCACAGTAGTGGCAAC-3’
**Igf1**	Igf1-Wt-F	5’-CAAATGTTGCTTGTCTGGTG-3’
	Igf1-Wt-R	5’-GTCAGTCGAGTGCACAGTTT-3’
	Igf1-Tg-F	5’-ACCAGAGGGAATTACTATAGC-3’
	Igf1-Tg-R	5’-TCTCCAGCCTCCTTAGATCAC-3’

Primer sequences for *Igf1 and Gsk3β* sourced from Jackson Laboratories genotyping protocols (jax.org).
Primers spanning *Twist1* Ser192Pro designed in-house.
